# Research Progress of Blood-Based Biomarkers for the Diagnosis and Prognostic Evaluation of Acute Ischemic Stroke

**DOI:** 10.3390/biom16070937

**Published:** 2026-06-24

**Authors:** Yuheng Shu, Yiren Qin, Qi Fang

**Affiliations:** 1 Department of Neurology, First Affiliated Hospital of Soochow University, No. 899 Pinghai Road, Suzhou 215006, China; 20245232072@stu.suda.edu.cn (Y.S.); qinsiming007@163.com (Y.Q.); 2 Department of Neurology, Dushu Lake Hospital, Suzhou 215006, China

**Keywords:** acute ischemic stroke, blood-based biomarkers, GFAP, neurofilament light chain, hemorrhagic transformation, neurovascular unit

## Abstract

Blood-based biomarkers offer a promising “biochemical imaging” approach for acute ischemic stroke (AIS) management, providing objective and accessible tools to complement conventional neuroimaging. This narrative review synthesizes recent advances in biomarkers derived from multiple neurovascular unit (NVU) compartments, including glial fibrillary acidic protein (GFAP), S100 calcium-binding protein B (S100B), ubiquitin carboxy-terminal hydrolase L1 (UCH-L1), neuron-specific enolase (NSE), neurofilament light chain (NfL), matrix metalloproteinase-9 (MMP-9), Claudin-5, Occludin, brain-derived neurotrophic factor (BDNF), interleukin-33 (IL-33), tumor necrosis factor-alpha (TNF-alpha), PARK7/DJ-1, glycogen phosphorylase BB (GP-BB), and circulating microRNAs. We focus on their stage-specific clinical utility across three scenarios: (1) ultra-early differentiation between ischemic stroke and intracerebral hemorrhage in prehospital and emergency settings; (2) dynamic prediction and monitoring of hemorrhagic transformation after reperfusion therapies; and (3) assessment of infarct burden, neurorepair potential, and long-term functional outcomes. Despite their promise, clinical translation remains hindered by assay platform heterogeneity, lack of standardized cut-off values, limited cost-effectiveness data, and insufficient prospective validation adjusted for key covariates such as age and renal function. We further discuss multi-marker panel construction, including strategies to address biomarker collinearity and overfitting. Future directions emphasize stage-specific panels, point-of-care testing devices, and artificial intelligence algorithms to advance precision medicine in stroke care.

## 1. Introduction

Stroke has now become one of the leading causes of disability and death worldwide, killing about 6 million people each year and placing a huge burden on families and society [1]. In the treatment of AIS, “time is brain” remains the core principle. Therefore, achieving ultra-early and accurate diagnosis, rapid differentiation of stroke subtypes, and accurate prediction of prognosis, especially hemorrhagic transformation (HT), are central to optimizing clinical outcomes and reducing disability rates.

Currently, the clinical evaluation of patients with AIS mainly relies on the National Institutes of Health Stroke Scale (NIHSS) and neuroimaging examinations (e.g., CT and MRI) [2,3]. However, these traditional tools have important limitations in practical application: NIHSS scores are susceptible to evaluator experience and have low sensitivity for posterior circulation stroke; although imaging can visualize lesions, CT has limited sensitivity for ultra-early AIS, and expensive equipment requirements and lack of continuous dynamic monitoring limit its use in prehospital emergency and low-resource settings [4]. In addition, imaging changes often lag behind pathophysiological damage at the tissue level.

In recent years, blood-based biomarkers have gradually become “biochemical imaging” tools in stroke management due to their objectivity, reproducibility, minimally invasive sampling, and feasibility for point-of-care testing (POCT). When a stroke occurs, various cells in the NVU, including neurons, astrocytes, endothelial cells, pericytes, and microglia, undergo necrosis, apoptosis, or activation in response to ischemia, hypoxia, and reperfusion injury, causing specific proteins or micromolecules to be released into the blood circulation [5].

This review aims to summarize research progress on stroke-related biomarkers. Unlike previous descriptions that mainly categorize biomarkers by function, this review focuses on indicators based on the pathophysiological damage mechanism of the NVU: glial injury markers (GFAP and S100B), neuronal and axonal injury markers (UCH-L1, NSE, and NfL), BBB integrity markers (MMP-9, Claudin-5, and Occludin), inflammatory and neurorepair markers (BDNF, IL-33, and TNF-alpha), and emerging biomarkers such as PARK7/DJ-1, GP-BB, and microRNAs. We analyze their clinical value in ultra-early differential diagnosis, HT risk warning after reperfusion therapy, and long-term prognosis assessment, and explore the potential of stage-specific multi-marker panels and artificial intelligence in precision stroke medicine.

Assay methodology is introduced here because platform characteristics directly affect interpretation of biomarker concentrations and cut-off values. Conventional enzyme-linked immunosorbent assay (ELISA) is widely available and relatively inexpensive but generally has lower analytical sensitivity. Single Molecule Array (Simoa) is a bead-based digital immunoassay that enables ultra-sensitive detection of low-abundance proteins such as GFAP and NfL, whereas electrochemiluminescence immunoassay (ECLIA) offers automated high-throughput measurement. Therefore, cut-off values generated on ELISA, Simoa, or ECLIA platforms should not be used interchangeably without assay-specific calibration.

## 2. Method

**Figure 1:**
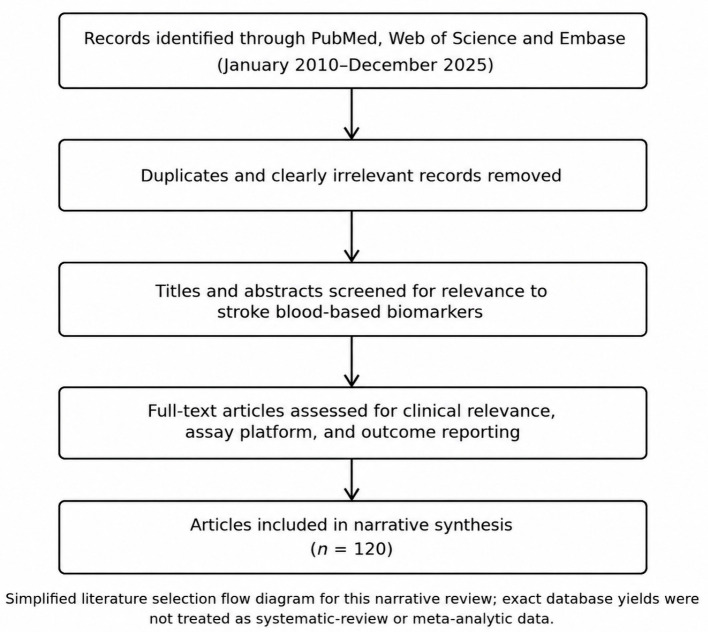
Simplified literature identification and selection flow diagram for this narrative review. The diagram summarizes database searching, screening, full-text assessment, and final narrative synthesis (*n* = 120) and is provided for transparency rather than as a formal PRISMA systematic-review flowchart.

A literature search was conducted in PubMed, Web of Science, and Embase for articles published between January 2010 and December 2025, using combinations of MeSH terms and free-text terms including “acute ischemic stroke”, “intracerebral hemorrhage”, “hemorrhagic transformation”, “blood biomarker”, “GFAP”, “S100B”, “UCH-L1”, “NSE”, “NfL”, “MMP-9”, “Claudin-5”, “CLDN5”, “Occludin”, “BDNF”, “IL-33”, “TNF-alpha”, “PARK7”, “DJ-1”, “NDKA”, “GP-BB”, “microRNA”, “miRNA”, “extracellular vesicles”, “ELISA”, “Simoa”, and “point-of-care testing”. Peer-reviewed original articles and reviews reporting on blood-based biomarkers in stroke diagnosis, HT prediction, or prognosis were considered. After screening titles, abstracts, and full texts, 120 relevant articles were synthesized in this narrative review. This review is a narrative synthesis rather than a systematic review or meta-analysis. To improve transparency, we added a simplified literature identification and selection flow diagram that summarizes the narrative search and screening process, without applying formal systematic-review reporting methodology (Figure 1).

## 3. Neurovascular Unit

The formal introduction of the neurovascular unit (NVU) concept dates to 2001, when it was articulated by the Stroke Progress Review Group of the National Institute of Neurological Disorders and Stroke (NINDS) [6]. The NVU is defined as a structural and functional entity, encompassing neurons, astrocytes, and microglia alongside vascular constituents including endothelial cells, pericytes, vascular smooth muscle cells, and the cerebrovascular basement membrane. The blood brain barrier (BBB), a critical substructure of the NVU, is anatomically composed of the capillary basement membrane, astrocytic end-feet, pericytes, and endothelial cells. In concert, these elements establish a dynamic interface that safeguards the brain against toxins, restricts the entry of harmful blood-borne chemicals, and mediates the controlled influx and efflux of nutrients essential for cerebral tissue.

**Figure 2:**
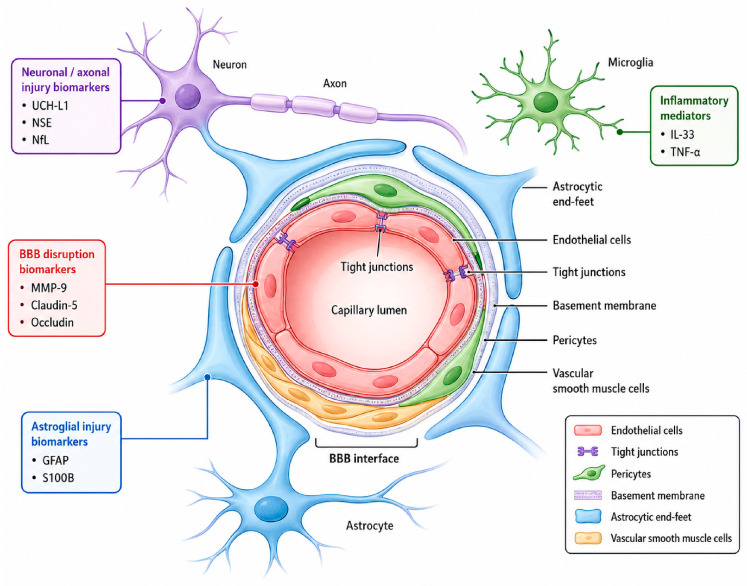
Detailed schematic of the neurovascular unit (NVU). The NVU includes neurons, astrocytes and their end-feet, microglia, endothelial cells with tight junctions, pericytes, vascular smooth muscle cells, and the basement membrane. Representative circulating biomarkers are mapped to the corresponding injury compartments: GFAP/S100B for astroglial injury, UCH-L1/NSE/NfL for neuronal and axonal injury, MMP-9/Claudin-5/Occludin for BBB disruption, and inflammatory mediators such as IL-33 and TNF-α.

The structural and functional integrity of the central nervous system (CNS) depends on the highly coordinated interactions between the various components of the Neurovascular Unit (NVU). The major structural components of the neurovascular unit are summarized in Figure 2. Within this framework, brain endothelial cells (ECs) represent a unique vascular interface characterized by the presence of tight junctions (TJs) [7]. These transmembrane protein complexes create a highly selective paracellular barrier that restricts the passive diffusion of solutes and inflammatory cells, governing transport based on molecular size, surface charge, and lipid solubility. Beyond structural containment, ECs maintain a dynamic regulatory mechanism for the bidirectional exchange of nutrients and metabolic waste between the systemic circulation and the neural parenchyma [8].

Complementing the vascular layer, pericytes (PCs) function as contractile mural cells embedded within the basement membrane of capillaries [9]. PCs are pivotal in modulating capillary diameter and regional cerebral blood flow [10]. Furthermore, they may serve as regulators of BBB stability and neuroinflammation by modulating immune cell trafficking [11]. Crucially, PCs contribute to post-stroke angiogenesis; their depletion or dysfunction triggers a downregulation of TJ proteins, leading to catastrophic BBB breakdown and vasogenic edema. Supporting the macrovasculature, vascular smooth muscle cells (VSMCs) regulate basal vascular tone and autoregulatory responses across the arterial tree.

Astrocytes, the most abundant glial population, exhibit remarkable structural and functional heterogeneity across brain regions [12]. Their specialized end-feet processes envelop the microvasculature, forming the glia limitans—the outermost layer of the BBB [13,14]. Astrocytes bridge the gap between vascular supply and neuronal demand, facilitating the transport of nutrients and neurotransmitters. Moreover, they actively modulate the neuroinflammatory milieu by secreting pro- and anti-inflammatory cytokines and chemokines, thereby orchestrating the response of microglia [15].

Microglia, the resident immune sentinels of the CNS, are dedicated to debris clearance and tissue repair following ischemic insult. Recent evidence suggests that microglia also interact directly with ECs to maintain BBB homeostasis. In turn, neurons communicate their metabolic requirements to other NVU components via the release of distinct signaling molecules and neurotransmitters. Finally, the basal lamina provides the structural scaffolding for the NVU [16,17]. This specialized extracellular matrix, composed of laminin, collagen IV, and heparan sulfate proteoglycans, is indispensable for maintaining the mechanical integrity of the cerebral vasculature.

### 3.1. Astroglial Injury Biomarkers

As the primary supportive scaffold of the NVU, astrocytes extend their end-feet to ensheath endothelial cells, forming the first line of defense for the Blood Brain Barrier (BBB).

#### 3.1.1. S100B

S100 calcium-binding protein B (S100B) is a glial protein expressed in mature perivascular astrocytes. This protein is involved in neurite elongation, astrocyte proliferation, and microtubule assembly inhibition. When neuronal damage or blood brain barrier disruption occurs, astrocytes are activated and release S100B into the bloodstream [18,19]. A study of 458 stroke patients showed that S100B had a sensitivity of 92.9%, a specificity of 48.1%, a positive predictive value of 12.2%, a negative predictive value of 98.9%, and an area under the curve of 0.746 [20]. Another study showed that the combination of S100B and IL-6 improved discrimination efficacy. The S100B level of ICH patients was significantly higher than that of IS patients (8 pg/mL vs. 4.2 pg/mL, *p* = 0.003), while the IL-6 level of IS patients was higher than that of ICH patients (12.9 pg/mL vs. 8.76 pg/mL, *p* = 0.02) [21]. This calcium-binding protein reflects astrocyte activation and early BBB leakage. Although its high sensitivity makes it a useful negative predictor for hemorrhagic transformation (HT), its clinical specificity remains limited due to its presence in extracerebral tissues.

#### 3.1.2. GFAP

GFAP is a cytoskeletal protein expressed primarily in astrocytes. It is not released under normal physiological conditions. Therefore, the blood level in healthy individuals is very low. When irreversible necrotizing damage occurs to brain tissue, such as the formation of an infarct core region, astrocytes disintegrate and GFAP is released in large quantities and crosses the damaged blood brain barrier into the blood circulation. In recent years, the value of GFAP as a biomarker for acute stroke has been extensively studied. Several multicenter studies have shown a rapid increase in GFAP in blood samples from patients with intracerebral hemorrhage compared to the slow release of ischemic stroke. Elevated serum concentrations of GFAP indicate a moderate sensitivity of approximately 75–90% and a specificity of >95% for ICH within the first few hours of symptom onset [22]. In early pioneering studies, Foerch et al. showed that within 6 h of symptom onset, the sensitivity and specificity of diagnosing cerebral hemorrhage at GFAP > 2.9 ng/L were 79%, 98%, and the positive predictive value was 94%, and the negative predictive value was 91% [23,24]. A study involving 213 patients showed that blood biomarkers serum NfL and GFAP measured in the acute phase of critical acute ischemic stroke improved the prediction of functional prognosis [25]. With a cut-off value of 0.7 ng/mL, GFAP had a sensitivity of 86% and a specificity of 76.9% for predicting intracerebral hemorrhage, and its level correlated with the degree of neurological dysfunction [26]. Another study showed that GFAP had a good ability to discriminate between clinical outcomes at the rate of change from admission to immediate vascular recanalization (pg/mL/h) (AUC = 0.88, *p* < 0.001) and was better predictive than CT-ASPECTS score at admission. Two meta-analyses confirmed that GFAP had a sensitivity of 75% to 78% and a specificity of 95% (AUC 0.9 to 0.93) for the combination of intracerebral hemorrhage and ischemic stroke or stroke-mimic presentations [27,28]. According to a study investigating the utility of serum GFAP and UCH-L1 in acute ischemic stroke, GFAP possesses the capability to discriminate between LVO and SVO subtypes, as well as between AIS patients and healthy controls [29]. Specifically, the higher concentrations of these markers were found in LVO patients, whereas healthy controls exhibited the lowest levels. The defined threshold for GFAP showed good sensitivity and specificity in differentiating LVO from SVO, and achieved excellent diagnostic efficacy when distinguishing LVO from healthy controls. Therefore, a combined assessment of GFAP and UCH-L1 could represent a valuable approach for differentiating LVO from SVO in AIS patients. Ongoing and future studies are expected to corroborate its role as an effective adjunctive tool for the early pre-hospital identification of AIS and for the rapid identification of patients who are appropriate candidates for endovascular therapy (EVT) [30].

**Figure 3:**
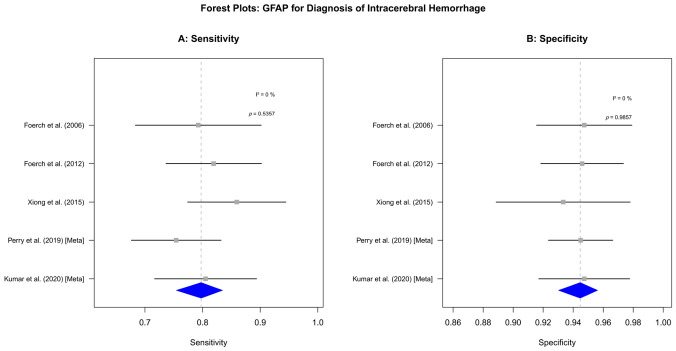
Forest plot of serum glial fibrillary acidic protein (GFAP) for the diagnosis of intracerebral hemorrhage (ICH), adapted from previously published studies [23,24,26,27,28]. (**A**) Sensitivity. (**B**) Specificity. Squares represent study-level point estimates, horizontal lines represent 95% confidence intervals, and blue diamonds represent pooled estimates.

The pooled sensitivity and specificity were 0.78 (95% CI: 0.71–0.84) and 0.95 (95% CI: 0.92–0.97), respectively. Significant heterogeneity was observed across studies (I^2^ = 82.3%, *p* < 0.001), which may be attributed to differences in assay platforms (Simoa vs. ELISA), cut-off values, and time from symptom onset to blood sampling. The area under the summary receiver operating characteristic (SROC) curve was 0.94 (95% CI: 0.91–0.96), indicating excellent diagnostic performance. Squares represent study-level point estimates, and horizontal lines indicate 95% confidence intervals. Data are pooled from previously published diagnostic accuracy studies. This figure is presented as a summary of existing evidence and does not represent a new meta-analysis conducted by the authors.The pooled diagnostic performance of GFAP across representative studies is summarized in Figure 3.

Despite the promise of GFAP, its clinical translation faces important challenges regarding diagnostic gain and assay standardization. Notably, Luger et al. [31] reported that adding UCH-L1 to a GFAP-based model did not significantly improve the overall AUC for differentiating acute stroke subtypes, highlighting potential redundancy among structurally related NVU-derived markers. In addition, cut-off heterogeneity remains unresolved: reported GFAP thresholds vary from 2.9 ng/L on ultra-sensitive Simoa-based platforms to 0.7 ng/mL on conventional ELISA-based platforms. This discrepancy is driven largely by analytical sensitivity and calibration rather than biological variance. Consequently, legacy ELISA cut-offs cannot be directly transferred to Simoa assays, and assay-specific validation is required before a universal clinical “red line” for ICH exclusion can be established.

### 3.2. Neuronal and Axonal Damage Indicators

Neurons are the most ischemia-sensitive components within the NVU, and their survival is the ultimate determinant of functional prognosis.

#### 3.2.1. UCH-L1

UCH-L1 is a highly abundant and specifically expressed enzyme in neurons for maintaining intracellular protein homeostasis. When acute, severe damage occurs to neurons, UCH-L1 rapidly leaks into the extracellular space and crosses the damaged blood brain barrier into the bloodstream. Therefore, studies have shown that serum UCH-L1 levels are thought to reflect the severity of acute neuronal body injury. In the past few years, the value of GFAP in the field of acute stroke has been well studied, but the clinical evidence on UCH-L1 is still insufficient. One study showed that GFAP and UCH-L1 can be used to quantitatively assess brain tissue damage and predict the prognosis of patients with intravenous thrombolysis. The cut-off values of GFAP and UCH-L1 were 116 pg/mL and 292 pg/mL, respectively, and the specificity for predicting 3-month adverse prognosis was 97.56% (95% CI: 94.51–99.00) and the positive predictive value was 88.68% (95% CI: 76.28–95.31) in the training cohort, and the specificity was 97.83% (95% CI: 91.62–99.62) and 96.90% (95%) in the test and validation cohort, respectively CI: 91.77–99.00), and the positive predictive values were 90.00% (95% CI: 66.87–98.25) and 75.00% (95% CI: 47.41–91.67), respectively. In addition, the nomogram model based on biomarkers showed good predictive power for 3-month prognosis in different cohorts [32]. For patients with intravenous thrombolysis, the level of UCH-L1 was 97.56% specific in predicting a 3-month adverse prognosis in the training cohort [33].

However, some studies have suggested that UCH-L1 does not significantly improve the performance of GFAP in terms of overall diagnostic accuracy [31]. The observation by Luger et al. that adding UCH-L1 did not significantly enhance GFAP-based prediction warrants a clinical re-evaluation of panel design. This lack of incremental value may suggest a potential ceiling effect for structural markers that mainly reflect NVU damage. Future panels may need to combine structural proteins with biomarkers from complementary pathways, such as systemic inflammation, BBB disruption, metabolism, or neurorepair, to capture biological signals that structural proteins alone cannot reflect. Therefore, the most valuable role of UCH-L1 may be as part of a carefully validated multi-marker panel rather than as a stand-alone prognostic marker [34].

#### 3.2.2. NSE

As an important metalloenzyme involved in glycolysis, neuron-specific enolase (NSE) is primarily expressed in mature neurons and cells of neuronal origin. Beyond its physiological role, NSE can also be produced by neuroendocrine tumors, including small cell lung cancer, where it serves as a useful differential diagnostic marker [35]. Upon neuronal injury or death, NSE is released into the systemic circulation; consequently, elevated blood levels of this enzyme have been correlated with larger stroke volumes. Therefore, monitoring NSE levels in stroke patients may help predict the risk of hemorrhagic transformation in acute stroke. One study showed that serum NSE was significantly elevated in patients with CVs such as posterior circulation ischemic stroke or vertebrobasilar artery insufficiency. This marker may serve as a differential diagnostic serology indicator for central and peripheral vertigo in patients with acute vertigo [36]. NSE dynamics are of great value in predicting hemorrhagic transformation: a study of 83 stroke patients found a significant increase in the risk of hemorrhagic transformation when NSE levels were elevated and a second peak occurred (odds ratio = 6.844) [37]. In summary, NSE is of great value in predicting bleeding and can distinguish central vertigo from peripheral vertigo. Despite NSE sensitivity, hemolysis can cause false positives, limiting its clinical reliability.

#### 3.2.3. NfL

NfL (neurofilament light chain) is a component of neurofilaments, the major intermediate filaments of axons. Its physiological function is to maintain axonal caliber, support intracellular communication between axons and dendrites, and indirectly regulate nerve conduction velocity, thereby conferring resistance to mechanical stress. NfL concentration is an indicator of axonal degeneration and can reflect central nervous system injury and neurodegeneration. The incremental rate of NfL may be a better predictor of prognosis than its absolute value. NfL levels are also associated with HT, which may help early detection and management of post-stroke complications. However, current studies are limited by the lack of external validation and limited sampling beyond two time points, which constrains assessment of NfL kinetics [38]. Another study showed that blood NfL levels were associated with clinical outcomes in moderate-to-severe ischemic stroke and with the occurrence of HT [39].

Furthermore, the combination of NfL with GFAP has been shown to improve functional prognosis prediction in severe AIS. Several studies have demonstrated that serum NfL and GFAP measured in the acute phase of severe AIS improve the prediction of functional outcomes [25]. Patients treated with mechanical thrombectomy combined with intravenous thrombolysis had significantly lower NfL concentrations (*p* = 0.0024) than patients who underwent mechanical thrombectomy alone. Group analysis by median biomarker level showed that patients with high NfL or high GFAP levels had less clinical improvement after treatment (as indicated by smaller changes in the 24 h NIHSS score), higher NIHSS scores during hospitalization, and higher mRS scores at 3-month follow-up [40]. In addition, one study showed that serum and urine NfL concentrations correlated most strongly in patients with normal renal function.

From a methodological perspective, the correlation between serum and urine NfL concentrations is strongest in individuals with normal renal function, highlighting the importance of considering renal clearance when interpreting NfL levels. Future studies with longitudinal sampling and external validation will be associated with defining the optimal kinetic parameters of NfL for clinical application.

### 3.3. Blood Brain Barrier (BBB) Integrity Indicators

The dynamic equilibrium between endothelial cells and the Basal Lamina is the cornerstone of the NVU’s barrier function.

#### 3.3.1. MMP-9

Matrix metalloproteinase 9 (MMP-9) is a zinc-dependent metalloproteinase involved in the degradation of the extracellular matrix. In stroke, the destruction of the endothelial layer leads to the release of molecules such as MMP-9, E-selectin, P-selectin, and von Willebrand factor, as well as exposure of the extracellular matrix. Elevated levels of MMP-9 in the blood are associated with hemorrhagic transformation after ischemic stroke [41,42]. In a study of 168 stroke patients, MMP-9 was 82.9% > 81.3% in predicting hemorrhagic conversion at 181.7 ng/mL, with a positive predictive value of 48% and a negative predictive value of 95.8% (sample collection was within 1 h of admission, all within 24 h of symptom onset) [43]. Kazmierski et al. found that MMP-9 had a predictive sensitivity of 68.7%, a specificity of 45.3%, a positive predictive value of 8.9%, and a negative predictive value of 94.9%. As a key mediator of extracellular matrix degradation, MMP-9 has high sensitivity and specificity in the prediction of post-stroke hemorrhage transformation, especially the negative predictive value, but its positive predictive value is relatively limited. Combining with other biomarkers can improve the ability of early diagnosis and classification of stroke [44].

#### 3.3.2. Claudin-5

Claudin-5, as a specific molecular marker of bloodbrain barrier integrity, has potential in the risk assessment of hemorrhagic transformation after ischemic stroke. An animal study showed that ischemic stroke reperfusion impaired the integrity of the bloodbrain barrier by down-regulating the tight junction protein Cldn5 (claudin-5), altering actin cytoskeletal adhesion, and high expression of the pro-inflammatory factor Il-6 (interleukin-6) [45]. Current research supports its use as a component of multi-marker prediction models (combined with S100B, MMP-9, etc.) rather than as a standalone diagnostic indicator. In the future, it is necessary to standardize the detection method and verify its predictive value in different stroke subtypes and treatment scenarios.

#### 3.3.3. Occludin

Occludin is a transmembrane tight-junction protein that contributes to BBB integrity. Compared with patients without clinically deteriorating HT, patients with clinically deteriorating HT showed higher levels of Occludin, S100B, and the Claudin-5/Occludin ratio, whereas vascular endothelial growth factor levels were lower. When assessed within 3 h of stroke onset, Claudin-5 was also associated with clinically deteriorating HT. These findings suggest that tight-junction proteins are more suitable for ED/stroke-unit monitoring during and after the intravenous thrombolysis window than for stand-alone prehospital decision-making.

### 3.4. Inflammatory and Neurorepair Pathway Biomarkers

#### 3.4.1. BDNF

BDNF is widely distributed in the central nervous system and belongs to the neurotrophic factor family, playing key roles in neuronal survival, differentiation, synaptic plasticity, and repair. It has attracted attention in studies of post-stroke recovery, neurodegenerative disease, mental disorders, and brain injury. One study showed that mean BDNF levels were significantly lower in stroke patients than in healthy controls (3.89 ± 2.05 ng/mL vs. 14.9 ± 4.7 ng/mL, *p* < 0.0001) [46]. BDNF can help distinguish stroke patients from controls but does not independently predict mortality. Baseline serum BDNF levels are also inversely associated with the risk of post-stroke depression after ischemic stroke [47].

#### 3.4.2. IL-33

Interleukin-33 is an immune-related cytokine involved in innate inflammatory responses [48]. One study showed that serum IL-33 predicted HT after AIS with an AUC of 0.739 (95% CI: 0.657–0.821, *p* < 0.001). When serum IL-33 was ≤67.66 ng/L, the sensitivity and specificity for predicting HT were 81.3% and 63.0%, respectively. Multivariate logistic regression analysis showed that serum IL-33 ≤ 67.66 ng/L was independently associated with HT after AIS (OR = 5.773, 95% CI: 1.685–19.792, *p* = 0.005). mRS follow-up results showed that the incidence of adverse functional outcomes in HT patients was significantly higher than that in non-HT patients [49].

#### 3.4.3. TNF-α

As a cytokine that orchestrates systemic inflammatory responses, TNF-α has been evaluated for its predictive value in the setting of carotid stenting. Specifically, when employed to forecast positive findings on diffusion-weighted imaging, this biomarker demonstrated modest diagnostic performance at a cutoff of 9.45 pg/mL, with 45.3% sensitivity, 82.8% specificity, and an AUC of 0.651 [50]. Higher serum TNF-α levels were significantly associated with new DWI lesions and IPH after carotid artery stenting, suggesting that TNF-α may be used as a potential biomarker for predicting acute ischemic brain lesions and IPH after carotid artery stenting [51].

### 3.5. Other Biomarkers

#### 3.5.1. PARK7/DJ-1

PARK7/DJ-1 is a protein that regulates oxidative stress responses and is upregulated in prostate tumors. Mutations in genes expressing PARK7 are also associated with autosomal recessive early-onset Parkinson’s disease [52,53,54]. Nucleoside diphosphate kinase is an enzyme responsible for catalyzing the exchange of phosphate groups between different nucleoside diphosphate groups. Following either ischemic or hemorrhagic brain injury, this enzyme exhibits upregulated expression. In one study, elevated levels of PARK7 and NDKA were detected as early as 3 h post-stroke. The diagnostic performance of these two biomarkers was reported as follows: for PARK7, sensitivity ranged from 54% to 91% and specificity from 80% to 97%; for NDKA, sensitivity fell between 70% and 90%, with a specificity range of 90% to 97%. Another study compared the levels of PARK7 and NDKA in different populations (Switzerland, Spain, and the United States) and showed that PARK7: 91% sensitivity and 80% specificity. NDKA: Sensitivity of 90%, specificity of 90% [55]. Neither biomarker showed the ability to distinguish between ischemic and hemorrhagic stroke and was not associated with the severity of brain injury [56].

#### 3.5.2. GP-BB

Glycogen phosphorylase BB (GP-BB) is an enzyme responsible for catalyzing the conversion of glycogen into glucose-1-phosphate. Under hypoxic conditions, this enzyme becomes activated alongside a marked reduction in glycogen breakdown. GP-BB is expressed in both cardiac muscle and brain tissue [57]. In the myocardium, it serves as an early biomarker of myocardial infarction, with plasma levels rising rapidly within one hour of chest pain onset in over 90% of affected individuals [58]. In contrast, within the brain, GP-BB is predominantly localized to perivascular astrocytes; consequently, disruption of the bloodbrain barrier results in an early elevation of its circulating levels. One study including 172 stroke patients and 133 non-stroke patients evaluated its potential as a biomarker for stroke diagnosis. Blood samples were collected within 4.5 h after symptom onset. With a cut-off value of 7.0 ng/mL, the sensitivity and specificity were 93%, and the area under the receiver operating characteristic curve was 0.96 [59,60]. To rule out elevated blood enzymes secondary to myocardial infarction, troponin was also tested.

#### 3.5.3. MicroRNA

MicroRNAs (miRNAs) regulate cellular activity by regulating the expression of downstream target genes and are involved in a variety of damage or repair processes. Due to their small molecular weight, miRNAs are able to be transported to the extracellular environment through extracellular vesicles. The bilayer lipid membrane structure of extracellular vesicles makes it easy to cross the bloodbrain barrier. When brain damage occurs, they can penetrate the injured area through surface proteins and lipid membranes, playing a key role in brain injury and repair. A study evaluated whether metabolomics could provide a potential diagnostic biomarker for stroke. With a sensitivity of 84% and a specificity of 76.9%, a panel comprising 11 biomarkers demonstrated the ability to differentiate between ischemic and hemorrhagic stroke. These biomarkers included hydroxybutyryl carnitine, glutarylcarnitine (C5DC), myristate carnitine, 3-hydroxypalmitoyl carnitine, hydroxystearyl carnitine, tyrosine/citrulline (Cit), valine/phenylalanine, C5DC/3-hydroxyisovalylcarnitine, C5DC/palmitoylcarnitine, ratios of total C0, C2, C3, C16, and C18:1 to Cit, as well as the propionylcarnitine/methionine ratio [61].

From a prognostic perspective, a systematic review and meta-analysis identified 68 miRNAs associated with post-stroke motor recovery and 7 miRNAs linked to cognitive recovery, with serum miR-9 and neutrophil-derived miR-29b emerging as the most promising prognostic candidates [62]. Furthermore, experimental studies have shown that miRNA-27a delivered via small EVs derived from cerebral endothelial cells promotes axonal remodeling by inhibiting Sema6A and RhoA, thereby improving functional recovery after ischemic stroke [63].

Collectively, these findings support the notion that circulating and EV-encapsulated miRNAs hold significant promise as non-invasive prognostic biomarkers for ischemic stroke. However, several challenges remain, including the lack of standardized protocols for EV isolation and miRNA quantification, small sample sizes in existing studies, and the need for large-scale prospective validation across diverse populations. Future research should focus on harmonizing detection platforms, establishing reference standards, and integrating miRNA signatures with clinical parameters and neuroimaging to develop robust multi-marker prognostic models.

## 4. Performance Evaluation in Clinical Application Scenarios

### 4.1. Ultra-Early Differential Diagnosis Between Prehospital and Emergency

**Figure 4:**
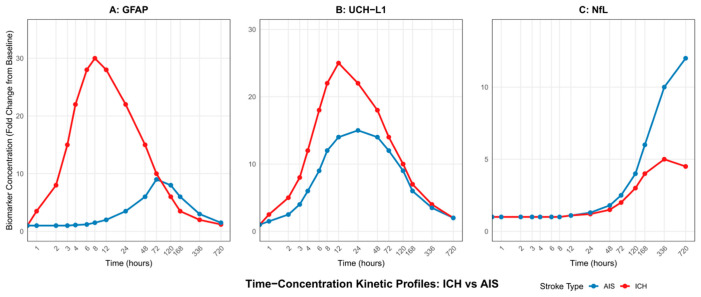
Time-concentration profiles of GFAP, UCH-L1, and NfL in ICH versus AIS. (**A**) GFAP kinetics. (**B**) UCH-L1 kinetics. (**C**) NfL kinetics.

One of the most important clinical applications of stroke biomarkers is rapid differentiation between ICH and AIS in prehospital and emergency settings, because this distinction directly affects thrombolytic eligibility. Figure 4 presents the time-concentration profiles of GFAP, UCH-L1, and NfL in ICH versus AIS. These kinetic patterns support a stage-specific approach: within the first 1–3 h after symptom onset, GFAP is the most useful blood marker for ICH rule-in because it rises rapidly after hemorrhagic astroglial disruption, whereas its elevation in AIS is usually delayed by slower BBB leakage. UCH-L1 may add information on acute neuronal injury, but its incremental value should be interpreted cautiously and validated against imaging. Stroke mimics remain an additional source of diagnostic uncertainty in emergency settings [64]. Importantly, slower markers such as S100B, MMP-9, NSE, Claudin-5, and Occludin should not be used as stand-alone biomarkers for ambulance thrombolysis decisions in the golden-hour window. Instead, prehospital testing should be considered an adjunct to clinical assessment and should be confirmed by non-contrast CT whenever available.

### 4.2. Thrombolysis and Mechanical Thrombectomy Decision-Making: Risk Warning and Monitoring

**Figure 5:**
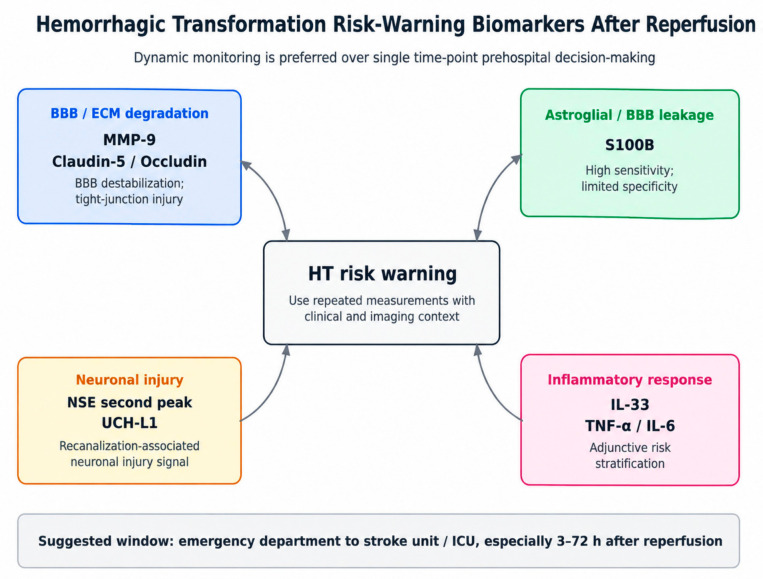
Hemorrhagic transformation risk-warning biomarkers and their monitoring roles after reperfusion therapy.

By contrast, prediction of HT after thrombolysis or mechanical thrombectomy is a dynamic monitoring task rather than a single prehospital triage decision. Figure 5 summarizes the main biomarker categories and monitoring roles for HT risk warning after reperfusion therapy. MMP-9 reflects extracellular matrix degradation and BBB destabilization, whereas S100B, Claudin-5, and Occludin reflect astroglial activation and tight-junction injury. These markers are most clinically relevant in the emergency department, stroke unit, or intensive care unit from the early post-reperfusion phase through the first 24–72 h, when repeated measurements can detect worsening BBB disruption [65]. NSE dynamics are also relevant because a second NSE peak after recanalization has been associated with a significantly increased risk of HT (OR = 6.844) [37]. Therefore, the proposed panel should be interpreted as a staged workflow: GFAP ± UCH-L1 for ultra-early subtype triage, followed by MMP-9/S100B/tight-junction proteins and NSE for post-treatment monitoring.

### 4.3. Disease Progression and Infarct Volume Prediction

Biomarkers also have predictive value for short-term prognosis over days to months after ischemic stroke. NSE has quantitative value because its levels correlate with infarct volume and neuronal loss; its peak concentration at 48–72 h after onset may provide additional information on the extent of neuronal injury, especially after large-vessel occlusion or reperfusion therapy [37]. S100B released from astrocytes is associated with BBB leakage, cerebral edema, and severe mass effect, but its interpretation is limited by low specificity and extracerebral sources. Therefore, NSE and S100B are better suited for adjunctive monitoring of disease progression than for initial stroke-subtype diagnosis.

### 4.4. Long-Term Prognosis Assessment and Disability Classification

Some biomarkers can also guide long-term prognostic assessment, including disability grading using scales such as the modified Rankin Scale (mRS). Unlike short-term fluctuations in GFAP, NfL reflects persistent axonal degeneration. NfL levels within 24 h to 7 days after onset have been associated with 3-month functional outcome, but their clinical validity requires adjustment for age, renal function, baseline stroke severity, and pre-existing neurodegenerative disease. BDNF may provide complementary information on neuroplasticity, post-stroke depression, and cognitive recovery, whereas circulating miRNAs may help characterize recovery-related molecular pathways [62,63,66]. A combined BDNF/NfL interpretation may therefore be useful for assessing neurorehabilitation potential, provided that models are externally validated and calibrated. The stage-specific clinical applications and interpretation caveats of major biomarkers are summarized in Table 1.

**Figure 6:**
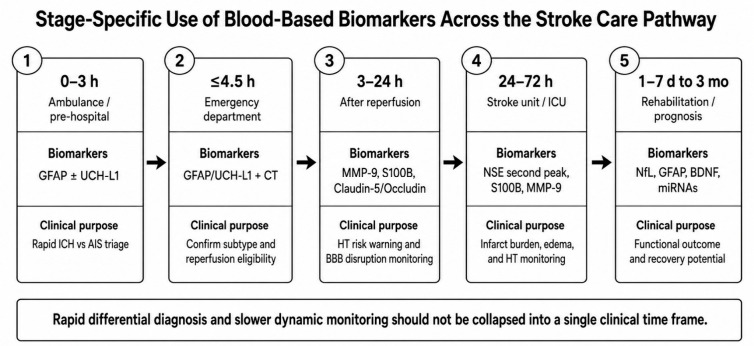
Proposed stage-specific biomarker workflow across the stroke care pathway. GFAP and UCH-L1 are prioritized for ultra-early triage, whereas MMP-9, S100B, Claudin-5/Occludin, and NSE are used for post-reperfusion monitoring; NfL, BDNF, and miRNAs are used mainly for longer-term prognosis. Numbers 1–5 represent consecutive stages of stroke care, and arrows indicate progression from acute diagnosis to long-term outcome assessment.

The clinical interpretation of these biomarkers must account for their distinct temporal kinetics and clinical setting. GFAP and UCH-L1 are most relevant for ultra-early subtype triage, whereas MMP-9, S100B, Claudin-5, Occludin, and NSE are better suited for dynamic post-reperfusion monitoring. NfL, BDNF, and miRNAs contribute mainly to subacute and long-term prognostic assessment. Figure 6 summarizes the proposed stage-specific workflow and clarifies that high-speed differential diagnosis and slower complication monitoring should not be collapsed into a single time frame.

## 5. Methodological Considerations for Multi-Marker Panel Construction

### 5.1. Multi-Marker Panel Construction

To address the inherent complexity of the NVU injury cascade, multi-marker panels should be constructed according to both pathophysiological complementarity and clinical timing. For example, GFAP and UCH-L1 represent astroglial and neuronal cell-body injury, respectively, whereas MMP-9 and tight-junction proteins represent BBB disruption, and NfL represents delayed axonal degeneration. Therefore, panels should not simply combine all available biomarkers in a single logistic model; instead, marker selection should match the intended clinical decision point, such as prehospital triage, post-reperfusion monitoring, or long-term prognosis.

The apparent inconsistency in reported correlations reflects differences in marker pairs and cohorts. Ren et al. reported a modest correlation between GFAP and UCH-L1 (r = 0.36, *p* < 0.01) [67], supporting partial non-redundancy for this specific pair. In contrast, stronger correlations between biomarkers originating from related NVU injury pathways may indicate possible collinearity. Therefore, model construction should include correlation matrices, variance inflation factor (VIF) assessment, and internal validation. If collinearity is present, penalized approaches such as LASSO or Ridge regression, dimension reduction, or pre-specified stage-specific marker selection should be preferred over simple unpenalized logistic regression.

Model performance should be evaluated using discrimination, calibration, and clinical usefulness rather than AUC alone. ROC analysis remains useful for comparing single-marker and multi-marker models, but calibration plots, HosmerLemeshow testing, decision-curve analysis, net reclassification improvement, and external validation are necessary to determine whether a panel provides clinically meaningful diagnostic gain. Age, NIHSS, renal function, time from onset to sampling, reperfusion status, and assay platform should be included as covariates or stratification factors, especially for NfL and GFAP models.

This integrative statistical framework underscores the value of combining pathobiologically aligned biomarkers to capture the multifaceted nature of ischemic brain injury, thereby improving early diagnostic precision and prognostic stratification in acute stroke settings.

### 5.2. Health Economic Considerations: Screening-Then-Confirm Strategy

In resource-constrained healthcare settings, integration of blood-based biomarkers into the diagnostic pathway for acute stroke warrants careful health economic evaluation. A proposed “GFAP-first, CT-confirm” strategy should be interpreted as a potential triage supplement for regions with limited imaging access rather than a replacement for non-contrast CT. The approach may be most relevant in rural or prehospital systems where CT is unavailable or delayed.

From a healthcare system perspective, a decision-analytic model can be constructed to evaluate the incremental cost-effectiveness of this sequential strategy compared with direct CT. Key model parameters include: (i) GFAP diagnostic accuracy for distinguishing ICH from AIS; (ii) ICH prevalence among suspected stroke populations; (iii) unit costs of GFAP testing and non-contrast CT; and (iv) clinical outcomes quantified in quality-adjusted life years or disability-adjusted life years. For practical cost reporting, Simoa-based GFAP testing has been estimated at approximately USD 50–80 per test (about CNY 360–580, depending on platform, reagent volume, and local reimbursement), whereas non-contrast head CT in Chinese hospital-cost data is approximately CNY 150–262 per scan in primary or acute-care settings [68,69]. These values should be regarded as approximate and should be replaced with institution-specific charges in formal economic models.

However, external validity remains a major concern: biomarker and CT costs vary substantially across healthcare systems, insurance models, laboratory infrastructure, and platform availability. Therefore, future studies should report costs in a defined currency and year, specify whether the estimate refers to reagent-only, laboratory service, or reimbursed clinical price, and perform sensitivity analyses across plausible cost ranges.

While direct evidence for such a strategy in stroke remains limited, analogous cost-effectiveness analyses in traumatic brain injury have demonstrated that biomarker-guided CT triage reduces unnecessary imaging by 30–40% and achieves net cost savings without compromising diagnostic accuracy [70]. Translating this framework to acute stroke care, a GFAP-first approach may be particularly beneficial in prehospital or rural settings where CT availability is limited, enabling more efficient resource allocation and potentially reducing time to appropriate treatment.

Collectively, these considerations underscore the need for formal health economic modeling to inform policy decisions regarding the adoption of blood-based biomarkers into routine stroke management pathways, particularly in low- and middle-income countries where cost constraints are paramount.

## 6. Challenges and Future Prospects: Toward Precision Medicine for Stroke 3.0

The transition of blood-based biomarkers from laboratory research to routine clinical practice faces several critical hurdles, yet also presents transformative opportunities for personalized stroke care.

### 6.1. Harmonization and Technical Standardization

One of the primary obstacles is the lack of analytical standardization. Current studies utilize diverse detection platforms, ranging from traditional ELISA to ultra-sensitive Single Molecule Array (Simoa) and electrochemiluminescence (ECLIA). This leads to significant inter-study variability in “cut-off” values. For markers like NfL, which exist in picogram levels, even slight variations in pre-analytical handling (e.g., sample storage, centrifugation speed) can skew results. Establishing international reference standards and participating in external quality assurance (EQA) programs is mandatory for achieving cross-platform comparability [70].

### 6.2. Optimizing the “Golden Hour”: POCT Development

In the hyper-acute phase, the clinical utility of a biomarker is inversely proportional to its Turnaround Time (TAT). To compete with the speed of non-contrast CT, the TAT must be reduced to less than 20–30 min. The development of Point-of-Care Testing (POCT) devices—utilizing microfluidics, lateral flow assays (LFAs), or electrochemical biosensors—is relevant. Integrating these devices into mobile stroke units (MSUs) or ambulances could shift the diagnostic window from the hospital to the field, facilitating ultra-early triage and treatment [71]. The vision of “Precision Medicine 3.0” relies heavily on Point-of-Care Testing (POCT). However, current turnaround times (TAT) are often bottlenecked by pre-analytical steps such as centrifugation and plasma separation. While electrochemical sensors show promise in laboratory settings, their deployment in ambulances remains speculative.

### 6.3. From Static Concentrations to Kinetic Modeling

A single “snapshot” measurement often fails to capture the dynamic evolution of the NVU injury. Future research should emphasize kinetic monitoring. By tracking the “rate of rise” or “peak time” of markers like GFAP and UCH-L1, clinicians can differentiate between transient ischemia and irreversible necrosis. Dynamic modeling allows for a real-time “molecular movie” of the stroke’s progression, enabling more nuanced therapeutic adjustments [72].

### 6.4. Multi-Omics Integration and Big Data

The complexity of the ischemic cascade suggests that no single protein can provide a complete picture. The future lies in Multi-omics integration, combining proteomics with transcriptomics (e.g., circular RNAs, microRNAs) and metabolomics. For instance, combining lipid metabolites (reflecting membrane degradation) with protein markers of axonal injury (NfL) could provide a holistic view of NVU health [73].

### 6.5. AI-Driven Personalized Prognostics

Artificial Intelligence (AI) and Machine Learning (ML) are poised to revolutionize biomarker interpretation. Unlike traditional linear regression, ML algorithms can process high-dimensional, non-linear data—integrating biomarker concentrations with clinical parameters (age, NIHSS, comorbidities) and multimodal imaging data (CT perfusion, MRI-DWI) [74]. This “Digital Phenotyping”, which uses digital data to characterize individual health behaviors and physiological states, may allow the development of personalized prognostic scores, identifying “fast progressors” vs. “slow progressors” and tailoring the intensity of post-stroke rehabilitation. Although current AI-based stroke prognostic models have achieved an AUC of 0.85–0.90, data on calibration and clinical usability remain scarce, posing risks for direct application in clinical decision-making.

The clinical translation of these findings is being actively tested in large-scale international initiatives. Projects such as the BIOSTROKE study and the PRESTO (Pre-hospital Stroke) consortium are currently validating multi-marker panels within real-world pre-hospital and acute settings. These efforts will provide the necessary evidence to move beyond ‘digital phenotypes’ and toward robust, AI-integrated decision support.

### 6.6. Challenges

Despite the considerable promise of blood-based biomarkers, their routine clinical application remains confronted with multiple challenges. A substantial gap exists between conventional laboratory-based detection and point-of-care testing (POCT), with current platforms—ranging from traditional ELISA and electrochemiluminescence to ultra-sensitive Simoa—exhibiting marked variability in sensitivity, leading to heterogeneous reported cut-off values. The absence of internationally standardized reference materials represents a major barrier to guideline incorporation. In the emergency setting, turnaround time (TAT) poses an additional hurdle, as conventional laboratory testing typically requires 2–4 h, often lagging behind neuroimaging in the ultra-early diagnostic window for acute ischemic stroke. Thus, the development of robust and highly sensitive POCT devices is a prerequisite for clinical translation [71].

Biomarker concentrations are further influenced by physiological and pathological covariates. Chronic kidney dysfunction may delay NfL clearance and cause spurious baseline elevation, while advanced age, pre-existing neurodegenerative diseases, systemic inflammation, infection, and renal insufficiency can independently alter GFAP, NfL, NSE, and inflammatory biomarkers. Consequently, reported AUC values for prognostic panels have limited clinical validity unless they are adjusted for age, renal function, baseline NIHSS, time from onset to sampling, treatment modality, and assay platform. This is particularly important for NfL-based 3-month outcome prediction.

Although the 2024 AHA/ASA Guideline for the Primary Prevention of Stroke has begun to recognize the potential of multi-omics approaches in stroke risk stratification and explicitly calls for the integration of artificial intelligence and big data to refine the evidence, guideline-level support remains lacking for acute-phase diagnostic and prognostic biomarkers such as glial fibrillary acidic protein (GFAP), ubiquitin carboxy-terminal hydrolase L1 (UCH-L1), and neurofilament light chain (NfL). This gap is primarily attributable to insufficient standardization of detection methods, high heterogeneity in reported cut-off values, and the absence of large-scale, prospective, multicenter validation studies. Future efforts should focus on bridging the evidence gap from biomarker discovery to clinical translation, including the establishment of international reference standards, the conduct of health economic evaluations, and the implementation of point-of-care testing (POCT)-based prospective clinical trials.

The development of multi-marker panels also faces statistical challenges. Correlated markers may increase variance inflation and overfitting if entered together into a simple logistic regression model, especially in small cohorts. Future studies should pre-specify the clinical time window, test multicollinearity using correlation matrices and VIF, apply penalized regression or machine-learning methods with nested cross-validation when appropriate, and report calibration, decision-curve analysis, and external validation. Only models that demonstrate incremental benefit after adjustment for clinical covariates should be considered candidates for clinical translation.

## 7. Conclusions

Future stroke management will shift from single-indicator interpretation to stage-specific multimodal fusion models. Ultra-early triage requires rapid markers such as GFAP with or without UCH-L1, whereas post-reperfusion monitoring requires dynamic assessment of BBB and neuronal injury markers such as MMP-9, S100B, Claudin-5/Occludin, and NSE. Long-term outcome prediction requires integration of NfL, BDNF, miRNAs, clinical scales, and neuroimaging. The ultimate goal is to build a comprehensive evaluation system covering stroke subtype, injury severity, complication risk, and repair potential. Such models must be standardized across assay platforms, adjusted for relevant covariates, validated prospectively, and evaluated for cost-effectiveness before routine clinical adoption.

## Figures and Tables

**Table 1:** Stage-specific clinical applications, kinetic windows, and interpretation caveats for key blood-based biomarkers in stroke.

Patient Encounter/Time Window	Clinical Question	Suggested Biomarkers	Primary Utility	Interpretation Caveats
Ambulance/prehospital (0–3 h)	Rapid subtype triage	GFAP ± UCH-L1	ICH rule-in and AIS/ICH differentiation	Adjunct only; false negatives may occur in small ICH; confirm with CT when available
Emergency department (≤4.5 h)	Reperfusion eligibility	GFAP, UCH-L1, clinical scales, non-contrast CT	Accelerate decision-making and reduce stroke-mimic uncertainty	Turnaround time should be <20–30 min; biomarkers should not delay standard imaging
Early post-reperfusion (3–24 h)	HT risk warning	MMP-9, S100B, Claudin-5, Occludin	BBB disruption and tight-junction injury monitoring	Best interpreted dynamically; not designed for single prehospital decisions
Stroke unit/ICU (24–72 h)	Infarct burden and deterioration	NSE, S100B, MMP-9	Monitor neuronal loss, edema, and secondary HT risk	NSE affected by hemolysis; S100B has extracerebral sources
Subacute to 3 months	Functional outcome and rehabilitation potential	NfL, GFAP, BDNF, miRNAs	3-month mRS, cognitive/affective outcome, neurorepair potential	Adjust for age, renal function, NIHSS, comorbidities, and assay platform
Panel design/research validation	Model construction	Stage-specific combinations	Improve discrimination beyond single biomarkers	Use VIF checks, LASSO/Ridge if collinearity is present, calibration, and external validation

## Data Availability

No new data were created or analyzed in this study. Data sharing is not applicable.

## Abbreviations

AIS	acute ischemic stroke
GFAP	Glial Fibrillary Acidic Protein
UCH-L1	Ubiquitin Carboxyl-Terminal Hydrolase Isozyme L1
NfL	Neurofilament Light Chain
BBB	blood–brain barrier
NVU	neurovascular unit
NSE	neuron-specific enolase
TNF-α	Tumor Necrosis Factor Alpha
ECs	endothelial cells
MMP-9	Matrix metalloproteinase 9
ICH	intracerebral hemorrhage
HT	hemorrhagic transformation
POCT	point-of-care detection
PCs	pericytes
TJs	tight junctions
CNS	Central Nervous System
S100B	S100 Calcium Binding Protein B
ELISA	Enzyme Linked Immunosorbent Assay
BDNF	brain-derived neurotrophic factor
PARK7/DJ-1	Parkinson Disease Protein 7 (Protein DJ-1)
IL-33	Interleukin 33
GP-BB	Glycogen Phosphorylase Isoenzyme BB
CLDN5	Claudin-5
OCLN	Occludin
